# A forgotten element of the blue economy: marine biomimetics and inspiration from the deep sea

**DOI:** 10.1093/pnasnexus/pgac196

**Published:** 2022-09-17

**Authors:** Robert Blasiak, Jean-Baptiste Jouffray, Diva J Amon, Fredrik Moberg, Joachim Claudet, Peter Søgaard Jørgensen, Agnes Pranindita, Colette C C Wabnitz, Henrik Österblom

**Affiliations:** Stockholm Resilience Centre, Stockholm University, 106 91 Stockholm, Sweden; Graduate School of Agricultural and Life Sciences, The University of Tokyo, 1-1-1, Yayoi, Bunkyo-ku, Tokyo 113-8657, Japan; Stockholm Resilience Centre, Stockholm University, 106 91 Stockholm, Sweden; SpeSeas, D'Abadie, Trinidad and Tobago; Marine Science Institute, University of California, Santa Barbara, CA 93106, USA; Stockholm Resilience Centre, Stockholm University, 106 91 Stockholm, Sweden; National Center for Scientific Research, PSL Université Paris, CRIOBE, CNRS-EPHE-UPVD, Maison de l'Océan, 195 rue Saint-Jacques, 75005 Paris, France; Stockholm Resilience Centre, Stockholm University, 106 91 Stockholm, Sweden; The Global Economic Dynamics and the Biosphere Academy Program, Royal Swedish Academy of Science, 104 05 Stockholm, Sweden; Stockholm Resilience Centre, Stockholm University, 106 91 Stockholm, Sweden; Stanford Center for Ocean Solutions, Stanford University, 473 Via Ortega, Stanford, CA 94305, USA; Institute for the Oceans and Fisheries, The University of British Columbia, 2202 Main Mall, Vancouver, BC V6T1Z4, Canada; Stockholm Resilience Centre, Stockholm University, 106 91 Stockholm, Sweden; Graduate School of Agricultural and Life Sciences, The University of Tokyo, 1-1-1, Yayoi, Bunkyo-ku, Tokyo 113-8657, Japan; South American Institute for Resilience and Sustainability Studies, CP 20200 Maldonado, Uruguay

## Abstract

The morphology, physiology, and behavior of marine organisms have been a valuable source of inspiration for solving conceptual and design problems. Here, we introduce this rich and rapidly expanding field of marine biomimetics, and identify it as a poorly articulated and often overlooked element of the ocean economy associated with substantial monetary benefits. We showcase innovations across seven broad categories of marine biomimetic design (adhesion, antifouling, armor, buoyancy, movement, sensory, stealth), and use this framing as context for a closer consideration of the increasingly frequent focus on deep-sea life as an inspiration for biomimetic design. We contend that marine biomimetics is not only a “forgotten” sector of the ocean economy, but has the potential to drive appreciation of nonmonetary values, conservation, and stewardship, making it well-aligned with notions of a sustainable blue economy. We note, however, that the highest ambitions for a blue economy are that it not only drives sustainability, but also greater equity and inclusivity, and conclude by articulating challenges and considerations for bringing marine biomimetics onto this trajectory.

## Introduction

Nature has been a source of inspiration for humanity throughout history (1, 2). Its influence is evident in the first tools and cave paintings, is reflected in histories, mythologies, and legends, and remains omnipresent today, inspiring everything from the design of airplanes and robots to computer algorithms, packaging, and corporate management structures (3–7). This is the world of biomimicry and biomimetic approaches, which seek to solve conceptual and design problems by mimicking or emulating the structure or performance of organisms and ecosystems that shape the natural world (8–10).

The starting point for biomimetics is the observation of the natural world. Yet the planet’s largest habitat—the ocean—has been mostly inaccessible for virtually the entirety of human history. For 3.7 billion years, life has existed in the ocean (three times as long as on land), and the tremendous variety of habitats in the ocean has resulted in comparably diverse morphologies, physiologies, and behavioral mechanisms (8, 11). While it covers 71% of the Earth’s surface, the vast majority of the ocean remains infrequently visited (12) and largely unseen. Deep-sea habitats in particular are among the least known on Earth (13). The deepest point in the ocean, the Challenger Deep in the Mariana Trench, was visited for the first time by humans in 1960—for a total of 20 min—and was not visited again for over 50 y (12, 14, 15).

Ocean exploration in recent decades has resulted in the discovery of deep-sea ecosystems where species have adapted to thrive under extremes of salinity, pressure, light, and temperature, including hydrothermal vents (1979) and brine pools (1983) (16–19). The potential for future discovery is vast: half of the ocean reaches depths of 4,000 m or more, only 20% of the seabed has been mapped (20), and 70% to 90% of marine species remain undescribed (19, 21). Real-time feeds from unmanned deep-sea submersibles outfitted with the latest camera equipment are now freely available (22–24), with thousands of people around the world sharing the experience of seeing unmapped parts of the seafloor and seamounts and unknown species for the first time. Museums around the world are filled with specimens and collections of deep-sea life (25), and thousands of genetic sequences (26), and even complete genomes (27–29), of deep-sea organisms are freely accessible in online databases (30, 31).

Recent advances in ocean science and exploration have occurred alongside a dramatic expansion in the scope and diversity of ocean-based activities and industries (32). Today’s ocean economy encompasses multiple industries that add up to over USD 1.9 trillion revenues annually (33), including industries as diverse as aquaculture, fisheries, oil and gas, offshore wind, cruise tourism, and marine biotechnology (34). As the ocean economy has grown, so too has attention to issues of equity and inclusivity, as just 100 companies—mostly headquartered in the Global North—accounted for nearly 60% of the ocean economy in 2018 (33, 35). In line with the UN Sustainable Development Goals and Agenda 2030, a variety of aspirational narratives have emerged of the ocean as a source of development that is not only sustainable but also equitable and inclusive. This type of ocean economy has been described by some as a “blue economy,” a term that has become widely used in recent years, but which remains without a broadly agreed definition (35, 36–40) (See note on p. 10). A complementary narrative of “exploration before exploitation” (41) underscores the loss that could accrue from embracing emerging extractive industries in the ocean, such as mining the seabed for metals and minerals, especially in the deep sea where recovery from impacts is extremely slow or impossible (42–44).

If mining and hydrocarbon extraction represent one approach to ocean resources, marine biomimetics represents a starkly different paradigm of resource use: innovation driven by exploration and understanding of the natural world and the life and processes that shape it. Yet in assessments of ocean uses and the blue economy, biomimetics is frequently excluded (33, 34, 45–48), or lumped together as a vaguely defined “emerging industry” (49, 50). We contend that marine biomimetics is a unique element of the ocean economy: vastly diverse and with key benefits for the viability of multiple industries; well-established rather than emerging; and worthy of greater attention in the context of aspirational narratives of a sustainable, inclusive, and equitable blue economy. The diversity of marine species that have spurred innovation also underscores the value of effective ocean conservation, as marine systems grow increasingly stressed by a changing climate and other anthropogenic pressures.

In the following, we first present a review of key focal areas for biomimetic design based on marine life, which then provides context for a closer look at the growing list of instances in which deep-sea life has inspired innovative design and technologies. We conclude by noting benefits that could arise from a more systematic articulation of marine biomimetics in the context of efforts to transform today’s ocean economy into a sustainable, inclusive, and equitable blue economy.

## Biomimetic design inspired by marine life

The study of marine life has yielded insights into a range of specialized adaptations that allow species to thrive in a diverse range of environmental conditions, and which have been a source of inspiration (8, 51). Categorizing the diversity of ways in which this inspiration translates into applications remains complex, with a wealth of associated terminology (Table 1), and often multiple nonexclusive terms used to describe a single innovation using a variety of available typologies (51–55) (e.g. by function, process, architecture, or material). For instance, the tubercles on humpback whale flippers (see the “Movement” section) have inspired the development of wind turbine blades, which are simultaneously “bio-inspired” (i.e. they are inspired by the structure of a natural material), “biomorphic” (i.e. they resemble the shape of a living thing), “biomimetic” (i.e. they mimic the structure of the flipper), and are also an example of “biomimicry” (i.e. they did not directly inspire the wind turbine, but rather a more sustainable and optimized design of the blades) (8, 56, 57).

**Table 1.** Key concepts associated with nature-inspired activities

**Concept**	**Description**	**Sustainability**	**Examples [inspired by]**	**Reference**
Biomimicry	Learning from and then emulating nature’s forms, processes, and ecosystems to create more sustainable designs	Main focus	Shinkansen 500 series bullet trains in Japan [kingfisher] (58), ventilation in Eastgate Centre, Harare [termite nest] (59)	Janine Benyus, 1997 (60)
Biomimetics	The process of mimicking the formation, structure or function of a biologically produced substance or material to produce or synthesize an artificial product. Derived from the Greek words *bios* (life) and *mimesis* (to imitate), biomimetics was coined by inventor Otto Schmitt (61, 62).	Frequently	Velcro [burdock] (63), gecko tape [gecko] (64), sharkskin swimsuit [sharks] (65)	Otto Schmitt, 1969 (62)
Bionic	The creation of modern engineering systems or a set of functions, based on biological systems and methods found in nature (or using artificial materials and methods to produce movement in a person or animal)	Rarely	Self-healing concrete [multiple organisms] (66, 67), implants in humans [axolotl] (68)	Jack Steele, 1960 (69)
Biophilia/biophilic design	Human tendency to interact or be closely associated with other forms of life in nature: a desire or tendency to commune with nature; biophilic design is an extension of biophilia	Important	Green or living walls inside offices; natural patterns, like curves and fractals, used in interior design [forests, meadows, waterfalls]	E.O. Wilson, 1986 (70)
Biomechanics	Study of the mechanical physics of biological processes or structures	Occasionally	Studying the aerodynamics of bird (71) and insect (72) flight and the hydrodynamics of swimming in fish (73)	Giovanni Alfonso Borelli, 1680 (74)
Bio-utilisation	The direct use of nature for ecological benefits	Occasionally	Gathering medicinal plants; growing algae to make biofuels; cultivation of *Artemisia annua* to produce malaria drug artemisinin (75)	Youyou Tu, 2011. (75)
Bioremediation	Use of microorganisms, plants, or enzymes, to detoxify contaminants in soil or other environments	Main focus	Using bacteria to break down oilspills (76, 77), or plants to bind, extract, and clean up heavy metals (78, 79)	Vidali, 2001 (80)
Biomorphic	Resembling or suggesting the forms of living organisms, often in design and art	Rarely	The Sagrada Família church by Antoni Gaudí [seashells, trees]; the citrus press “Juicy Salif” by Philippe Starck [squid] (81)	Geoffrey Grigson, 1935 (82)
Bio-affiliation	The idea that humans feel better and are healthier when in contact and connected with nature	Social sustainability	Therapeutic gardens for the elderly; parks in urban areas promoting physical activity [groves, meadows, streams]	Roy Remme et al, 2021. (83)
Bioinspired	General description of several of the concepts in this table, but also a way to emphasise that the resulting idea or innovation is not about simply copying nature	Context dependent	See all above [multiple]	Julian Vincent et al, 2006 (84)
Nature-based solutions	Actions that are inspired and supported by nature, seeking to build resilience while providing social, environmental and economic benefits.	Main focus	Tree-planting, coastal zone restoration [forests, mangroves, coastal dunes]	Alexandre Chausson et al, 2020 (85)

**Fig. 1.**
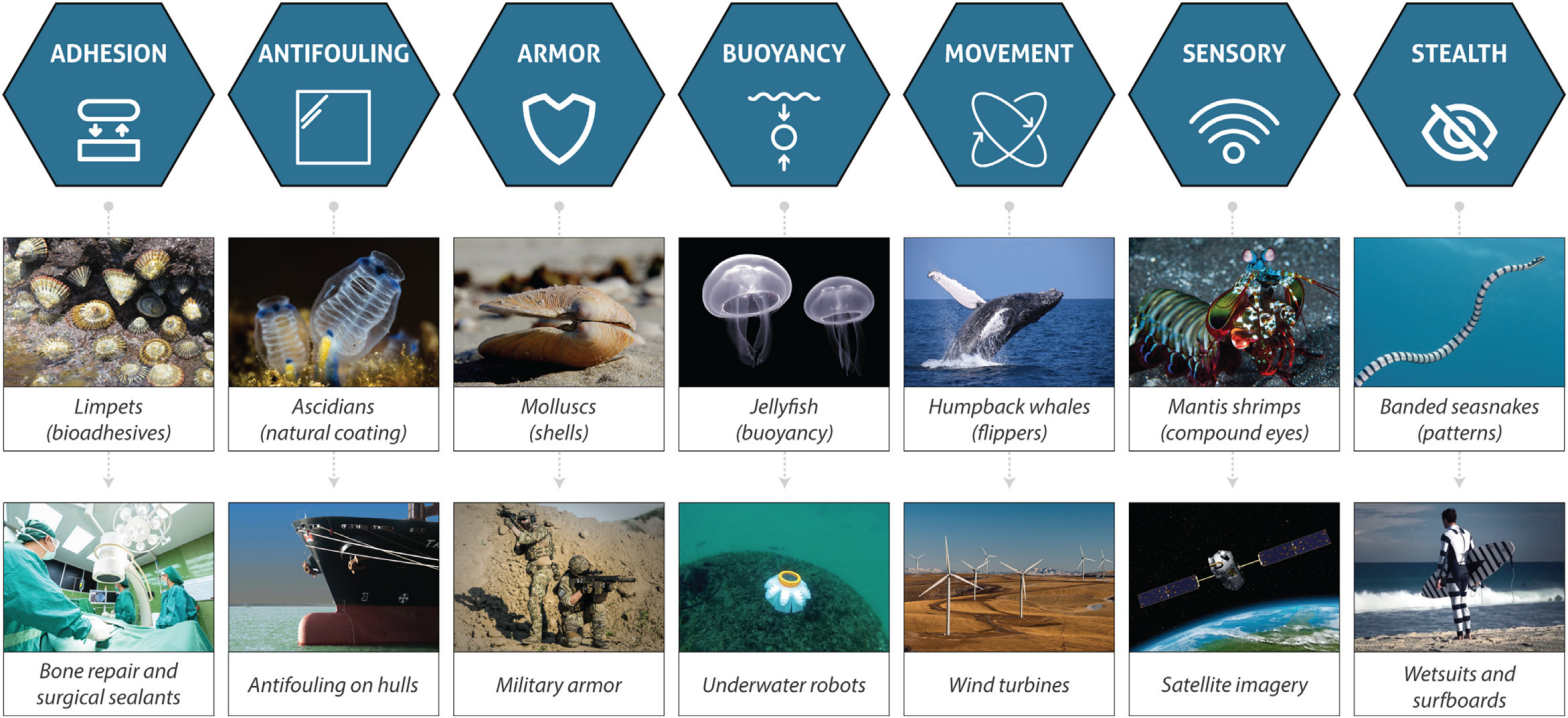
Marine biomimetics. The diverse morphological, physiological, and behavioral characteristics of marine species have inspired innovations that extend across diverse industries. Photo credits from left to right, first row: NOAA; Christian Gloor; CC0 Public Domain; Luc Viatour; CC0 Public Domain; Cédric Péneau; Christian Gloor. Second row: CC0 Public Domain; CC0 Public Domain; CC0 Public Domain; Jennifer Frame/JenniFish Ocean Test; CC0 Public Domain; NASA; SAMS.

In this Review, we follow the spirit of Otto Schmitt, who is credited with coining the term “biomimetics” in the 1950s and argued that biomimetics was simply the “transfer of ideas and analogues from biology to technology” (2, 61). A similar instinct for simplification was articulated by Vincent and co-authors, who suggested that biomimetics could be used synonymously with ‘“biomimesis,” “biomimicry,” “bionics,” “biognosis,” “biologically inspired design,” and similar words and phrases implying copying or adaptation or derivation from biology’ (2). While recognizing the potential for further disambiguation, we opt for this broader framing of biomimetics, and introduce seven broad groupings of marine biomimetic applications (Fig. 1).

### Adhesion

The survival of many marine organisms depends on their capacity to adhere to underwater surfaces. The biomineralized adhesives produced by barnacles and oysters as well as the adhesive “byssus threads” used by mussels allow for permanent adhesion, while viscous adhesive proteins secreted by echinoderms like sea stars, sea cucumbers, and sea urchins allow for temporary adhesion, locomotion, and handling of food (10, 86–90). The bioadhesive glue of limpets are up to 97% water, yet are comparable in strength to the cements of oysters and barnacles, and the diversity of marine invertebrate and diatom species that produce bioadhesive gels represent a vast research frontier (86, 88, 91, 92). The biomimetic potential associated with understanding the structure and chemistry of marine bioadhesives has applications across diverse medical fields focused on bone repair (10, 93), dentistry (94, 95), tissue engineering (96), and as surgical sealants (97–99), as well as in the construction of vessels and facilities in the marine environment, particularly when these require coatings and paints that need to adhere to water-facing surfaces (100–104). Similarly, proteins in the byssus threads that mussels use to attach to surfaces have inspired the development of adhesives, which are infused with cerium-oxide nanoparticles (105) to provide anticorrosion properties when applied to metal surfaces (106, 107).

### Antifouling

Wherever solid surfaces are found in the ocean, marine organisms begin to adhere to them, using the whole suite of chemical and structural adhesive capacities described in the previous section. This is a process called biofouling, namely the unwanted accumulation of such organisms to everything from submarine hulls to the cooling water intake pipes at nuclear power plants, and the implications for marine industries are severe. Biofouled ships, for instance, consume more fuel due to greater weight and increased friction, and potentially transfer invasive species across oceans (108, 109). The economic losses associated with marine biofouling currently cost marine industries over USD 150 billion annually (108, 110, 111), with estimates of the global market for marine coatings predicted to top USD 15 billion by 2024 (108). Conventional biocides and antifouling paints used for antifouling carry a heavy environmental impact, including bioaccumulation of organotins and copper in marine mammals and other marine life (112, 113). Biomimetic approaches to address these issues have focused in particular on the natural coatings and compounds produced by marine organisms (108, 114, 115), including ascidians (116), macroalgae, (117) algal compounds (118), marine bacteria (119, 120), and sponges, presumably in an effort to avoid biofouling themselves (121). Another rich source of inspiration has been the development of biomimetic surfaces based on the microtopography of marine organisms that function as natural antifoulants (115), including common marine shells, (122) crustaceans (123), seaweed (124), and sharkskin (125, 126).

### Armor

The scales of fish have evolved to provide multiple benefits, most notably providing armoured protection without sacrificing flexibility. The scales of fish (and snakes) were likely already inspiring the development of armors in human antiquity, and Ehrlich notes historical examples extending back to the time of the Persian and Roman empires in which scale armor is referenced or depicted (86, 127). The flexibility and protective properties of scales continue to inspire armor designs today (128, 129). The shells of mollusks have also inspired biomimetic designs [including military armor (130, 131)], with their nacreous layers outperforming conventional ceramics with regard to toughness and both tensile and compressive strength (132). The biomineralization process through which shells form has been the subject of substantial research (133–135), and multilayered ceramics and composites inspired by seashell nacre are in development (132, 136). Mantis shrimps, a frequent source of inspiration (also see the “Sensory” section), possess a pair of appendages called dactyl clubs that they use to strike prey at speeds comparable to a fired bullet with a force of up to 153 kg (although mantis shrimps themselves only weigh 12 to 90 g) (137). The periodic helical structure of the layered fibers in mantis shrimp dactyl clubs and lobster claws have been studied and resulted in bioinspired fiber composite laminates with increased resistance to impact force, denting, and cracking (138, 139).

### Buoyancy

**Fig. 2.**
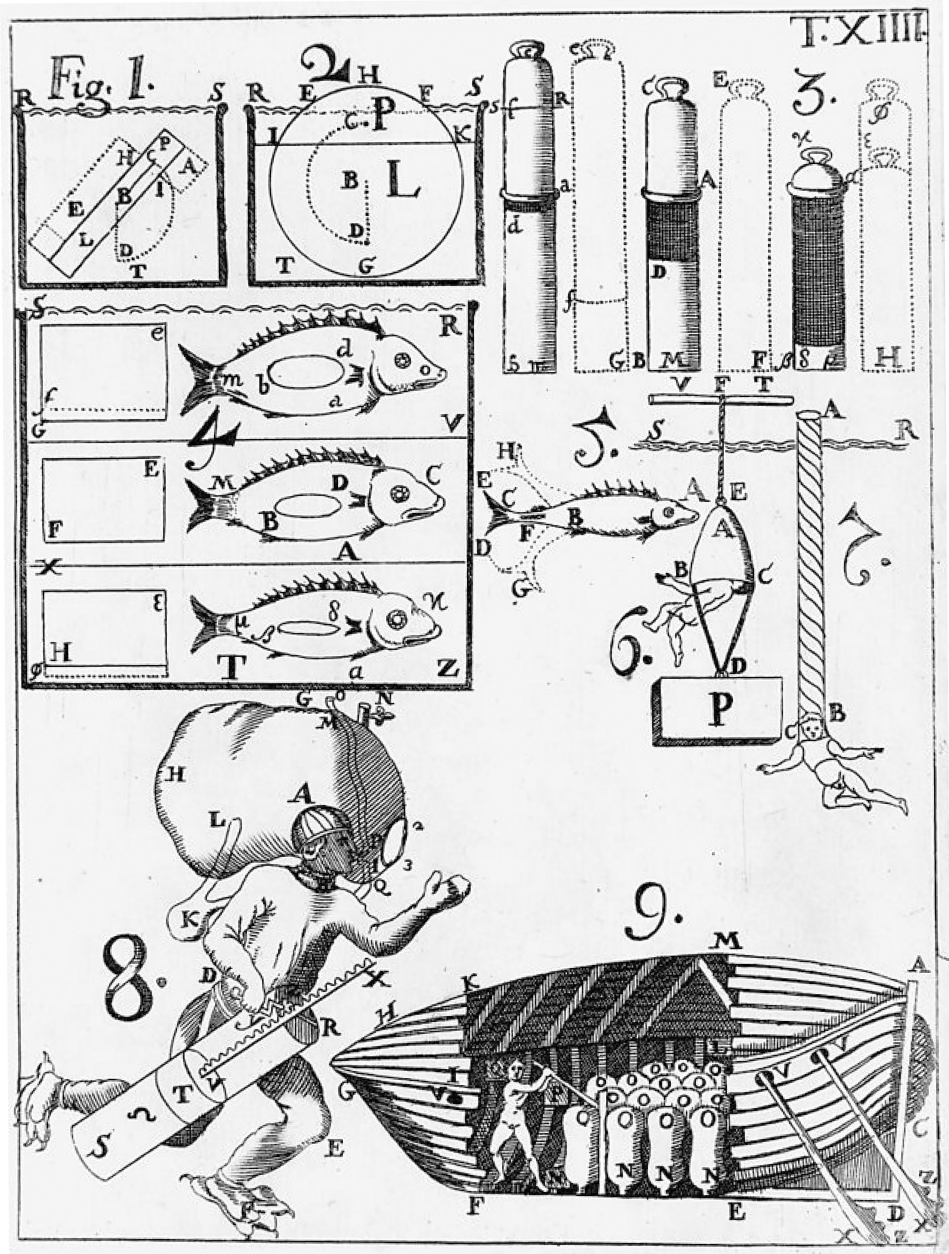
Panel from “On the Motion of Animals” (*De Motu Animalium*) by Giovanni Alfonso Borelli (1680) illustrating the properties of fish swim bladders (upper left), and how they have inspired biomimetic design of a submarine using air-filled goatskin bags (lower right) as well as a diver’s buoyancy device and tank of air (lower left) (74).

Neutral buoyancy can be an important asset for movement underwater (140–142), and led to the evolution of swim bladders in fish some 400 million years ago (51). These gas-filled sacs are flexible and adjustable, with fish equilibrating them to provide neutral buoyancy at the topmost reaches of their respective habitats (51, 143). Biomimetic design based on the swim bladder can be found during the Renaissance, with Giovanni Borelli publishing drawings of what could have been the world’s first submarine in 1680 (8, 74) (although no evidence exists that this project moved beyond conceptual drawings). Borelli’s illustrated plate (Fig. 2) includes not only biomimetic use of goatskin bags as a hydrostatic mechanism to submerge the submarine, but also a conceptualized diver who can move underwater using a large goatskin bag that was meant to double as a source of air and an adjustable buoyancy device (74). The latter innovation foreshadowed a modern-day buoyancy compensation device, that itself mimics the fish’s swim bladder, and enables divers to closely control their buoyancy (51). Buoyancy is a ubiquitous concern for operations at sea and underwater, and today there are a number of underwater robots, autonomous gliders and submersibles that have drawn inspiration not just from fish and their buoyancy-related movements (144), but from marine species as diverse as whales (145, 146), dolphins (147, 148), jellyfish (149–151), lobsters (149, 152, 153), and copepods (154).

### Movement

The movement of life below water has been perhaps the richest—and one of the most varied—sources of inspiration for biomimetic design. During the Renaissance, Juliana Berners (15th century) and Leonardo da Vinci (16th century) were already remarking on the movement of water eddies, with the latter noting how the streamlined shape of fish could reduce drag (8, 155, 156). The fusiform design of some fish, with rounded heads and bodies that gradually taper back to the tail, inspired (unsuccessful) biomimetic boat design efforts in the early 19th century based on close studies of the movements of dolphins and trout, and, much later, (successful) design of nuclear submarines (8, 157). While the shape and movement of dolphins continue to be a source of inspiration (147, 158), finned fishes too have drawn intense interest (159–161), as have sharks (162, 163) and rays (164, 165). The fluid mechanics associated with rounded tubercles on the flippers of humpback whales have inspired design both below and above water, most notably in the shape of wind turbines, tidal turbines, and even surfboards (56, 57, 166, 167), while the flexible waving of macroalgae has led to the development of kelp-inspired wave energy generators (168). The body design and propulsive systems of other marine life have inspired additional libraries of biomimetic design, including the jet propulsion and shape of squids (51, 169–171) and other mollusks (172), the movement of siphonophores (173, 174), and the bell shape and contractions of jellyfish (51, 151, 169, 175–177). Applications extend from underwater vehicles all the way to the design of robots and spaceships specially adapted to explore other planets with starkly different atmospheric and gravitational conditions (178). Recent advances in experimental modeling methods have even rendered the shape and movement of extinct marine animals such as plesiosaurs a source of rich biomimetic inspiration for the design of underwater robotics (179–184).

### Sensory

The sensory environment under water is distinctly different from above, with water rapidly absorbing light, and currents dispersing chemical trails (51). Consequently, marine life possesses sensory capacities particularly adapted to this environment. Elephant seals, for instance, vibrate their facial whiskers to help locate and pursue prey (185), while dolphins and some whales are able to produce high-frequency sounds that enable them to echolocate prey (51, 186, 187). Dolphins and humpback whales also famously use bubble nets to gather their prey into confined spaces, and while traditional sonar fails in environments with heavy sedimentation or bubbles, they still find their prey (51, 188, 189). Biomimetic work on developing dolphin-inspired sonar and radar is ongoing, leading to the development of sonar movement tags for tracking animal movements (190), with vast potential implications for maritime navigation (191). Another novel sensory adaptation is found in sharks, which use their electrosensory system to detect the weak electric field generated by fish. Sharks also possess a “cerebellum chip” that identifies their own electric field and is able to account for this when processing external electromagnetic signals (51). Both the system of electroreceptors and the shark’s ability to process this data have inspired the development of various innovations including sensors and artificial electroreceptors (51, 192, 193). A final sensory adaptation of note in the marine world is optical, as exemplified perhaps most spectacularly in the world’s 450 species of mantis shrimp. Among other things, mantis shrimp eyes are compound, can move independently of one another, rotate up to 70 degrees in any direction, and see 16 channels of color (humans can process three) as well as ultraviolet and polarized light (194). Associated biomimetic work has focused on adaptively enhancing contrast vision (195), developing novel photodetectors (196), and enhancing satellite imagery (197).

### Stealth

Marine life has evolved a diverse range of stealth adaptations, including passive camouflage that mimics background environment, and translucency that renders species almost invisible (51). Some species seek to mimic the appearance of other species that are poisonous, an approach also used in the design of banded surfboards and wetsuits meant to deter shark attacks by mimicking the patterns of banded seasnakes (51, 198). The active camouflage used by cephalopods like octopi and cuttlefish to opportunistically mimic substrates has drawn intense interest (199) due among other things to its rapidity, with chromatophores that can flash different colors roughly five times every second (51). Cephalopod chromatophore cells have inspired the development of artificial skin (200, 201), paint-like coatings that can be triggered to change color (202, 203), and artificial chromatophores (204, 205).

### Other applications

While the previous subsections showcase some of the broader categories of marine biomimetics, they are pieces of a vibrant and rapidly expanding discipline. A comprehensive list would include the many biomimetic designs based on chitin (206–209) and collagen (210–212), design and automation based on the schooling behavior of fish (213, 214), architecture inspired by marine species (215), electrochemical batteries inspired by electric rays (51, 216), use of coral skeletons as bone tissue scaffolds (217–220), and many other innovations. In an even broader sense, groups of organisms and even entire ecosystems can be a source of inspiration, as in the case of coral reefs serving as an inspiration for industrial symbiosis (221) or nature-based solutions (Table 1) such as artificial reefs and coastal protection measures (222, 223).

Looking further to the periphery of marine biomimetics, two additional categories of innovation are of interest. The first of these, marine biodiscovery, often extends beyond biomimetics into the world of bioutilization, with marine natural products (secondary metabolites produced by marine organisms) being collected and subsequently used or modified to produce both medical and nonmedical products. Some 700 new marine natural products are being discovered on an annual basis, and a total of nearly 40,000 have been identified to date (224, 225). In some cases, the proteins associated with marine natural products provide direct inspiration for the development of novel synthetic constructs. Iconic examples of commercial products originating from marine biodiscovery include a suite of marine drugs (20 or which are in clinical use today (226), while at least 33 more are in clinical trials) (227–229). The rates of successful drug discovery from marine natural products is up to four times higher than their terrestrial counterparts, suggesting rich further potential (230, 231). Looking beyond the specific instance of marine drugs, the study of marine life has been crucial for fundamental breakthroughs in medical science and beyond, including for instance the discovery of green fluorescent protein in the jellyfish *Aequorea victoria* (used for broad range of medical and biological applications) (232, 233), and understanding the cell cycle through experiments on sea urchins (and subsequent discovery of cyclins and associated drugs) (234). Collectively, 13 Nobel Prizes in chemistry and medicine have been awarded for work on marine organisms (235).

The second category of innovations located on the periphery of marine biomimetics does not look at individual organisms and ecological systems, but rather the conditions that create their environments, namely geomimetics (material design inspired by natural geological syntheses and natural materials formation) (236). An extreme case under this umbrella is geomimetic design based on hydrothermal synthesis (i.e. methods for crystallization under conditions of high pressure and temperature), although analogue systems exist not only in the ocean (e.g. hydrothermal vent systems, which can be found in both the shallow and deep sea), but also hot acidic springs on land (236). Hydrothermal synthesis has been used to generate both inorganic materials such as zeolite and synthetic gemstones, as well as organic “green” polymers (237) and polyimide-based covalent organic frameworks, which are promising materials for use as anodes in lithium-ion batteries (236, 238).

## The deep sea as a source of inspiration

From an anthropocentric perspective, the deep sea is a place of extremes: the largest biome on Earth, where sunlight does not penetrate, where pressures force us into thick-walled submersibles, and with a diversity of environments characterized by extremes (239). But what is extreme to us is attractive to others, with entire classes of organisms that thrive in extremes of temperature (thermophiles), acidity (acidophiles/alkaliphiles), salinity (halophiles), pressure (barophiles), and high metal concentrations (metalophiles) (236, 240). All such conditions are present in the deep sea, and often in combination, providing habitats for polyextremophiles (microorganisms that benefit from environments characterized by multiple of these “extreme” conditions) (236, 241).

Wherever we have looked for life in the deep ocean, we have found it, with over 25,000 deep-sea species already included in the World Register of Deep-Sea Species (WoRDSS), and likely orders of magnitude more yet to be described (11, 19, 242). While various depths have been suggested as the starting point of the “deep ocean” (243), here a 500 m threshold is considered in line with the WoRDSS database (239, 242). In contrast to the “Biomimetic design inspired by marine life” section, which provides a brief review of broad categories of marine biomimetics, the following subsections focus on individual deep-sea species and species groupings that have been a source of inspiration for diverse applications.

### Deep-sea fishes

#### All the better to see you with: the brownsnout spookfish and glasshead barreleye fish

**Fig. 3.**
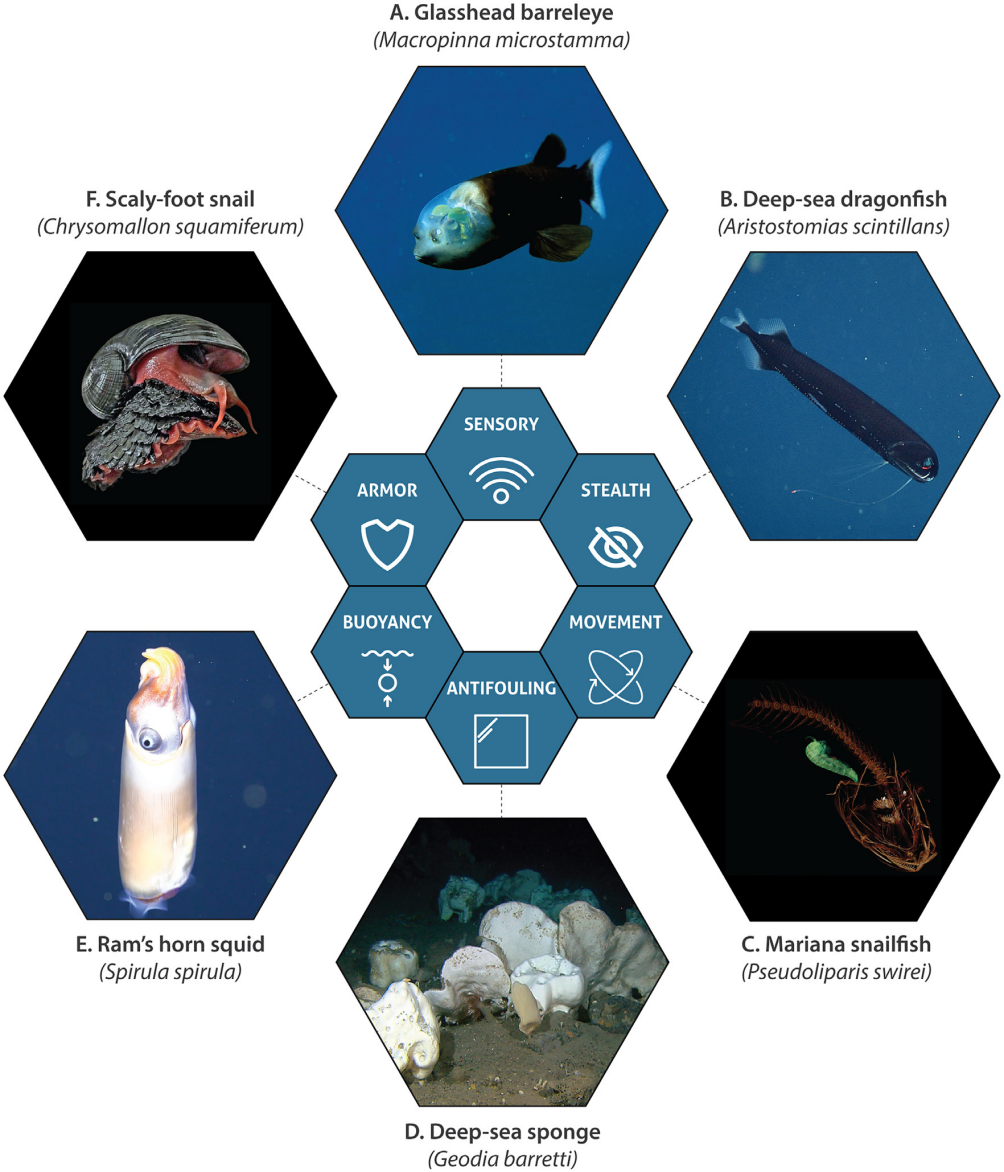
Inspiration from deep-sea species. Life in the deep sea is specially adapted to thrive in areas characterized by high pressure, absence of sunlight, and limited nutrients. Examples include (A) the mirrored eyes of mesopelagic teleost fish like the glasshead barreleye fish; (B) the transparent teeth of the deep-sea dragonfish; (C) the morphology of the Mariana snailfish (note the CT scan revealing a small crustacean, the green shape, in the snailfish’s stomach); (D) antifouling compounds extracted from deep-sea sponges; (E) the air-filled shell that provides buoyancy to the Ram’s horn squid; and (F) the armored shell of the Scaly-foot snail. Photo credits: (A) and (B) MBARI; (C) Adam Summers/University of Washington; (D) CoralFISH/Havforskningsinstituttet; (E) Schmidt Ocean Institute; (F) Chong Chen.

No light extends beyond 1,000 m into the ocean, but some faint traces of residual daylight persist at depths from 500 to 1,000 m, and a range of species in this zone produce bioluminescence (244) creating a unique sensory environment. A number of mesopelagic teleost fishes have evolved unique eyes that benefit from both sources of light (through a combination of reflective and refractive optics) (Fig. 3A), with the two most-studied species being the brownsnout spookfish (*Dolichopteryx longipes*) and the glasshead barreleye fish (*Rhynchohyalus natalensis*) (244–247). The reflective eyes of decapods like lobsters spurred advances in the field of astrophysics, namely the development of “lobster-eye” X-ray telescopes (248, 249), and the biomimetic design potential of further telescope innovations focused on the unique tilting of mirror plates in *D. longipes* eyes has been suggested (250, 251). A recent study created advanced models to test the optical performance of *D. longipes*, paving the way to the development of optical systems that can function in harsh environments including the deep sea (252). Other examples of biomimetic design are found in architecture, where the mirrored eyes of *D. longipes* inspired concepts of energy-saving roof designs by making optimal use of available daylight (253, 254), as well as in the design of a streamlined passenger car based on the body line of *R. natalensis* (255).

#### All the better to eat you with: the deep-sea dragonfish

While the structural properties of marine species have inspired the development of novel ceramics and related materials, such efforts have often focused on the nacreous layers of (coastal) mollusk shells. In the deep sea, the dragonfish (*Aristostomias scintillans*) initially attracted study due to its uniquely transparent teeth, which are thought to be an adaptation enabling further stealth, with the teeth becoming virtually invisible even in proximity to light produced by bioluminescent species (Fig. 3B) (256). A materials science approach to understanding *A. scintillans* teeth found that their transparency arises from a unique nanoscale structure, which also contributes to levels of hardness and sharpness comparable to the teeth of piranhas and great white sharks (256) and a source of inspiration for researchers looking to develop transparent ceramics (257).

#### Moving under pressure: the Mariana snailfish

The morphology and movement of dolphins and trout have inspired vessel design in shallow waters. Likewise, the development of vessels that can move in the deep sea benefits from close study of deep-sea life. In 2017, scientists discovered a new hadal snailfish species (*Pseudoliparis swirei*) (258) in the Mariana Trench and collected specimens at depths of over 6,000 m (29) (Fig. 3C). A close study of its morphology in 2019 (29) contributed to the spectacular development of an untethered soft robot with multiple points of inspiration from *P. swirei*, including the use of thin, flapping pectoral fins, and the distribution of comparatively heavier electronics within the robot’s “head” similar to the distributed weight of the snailfish’s skull (259). The soft robot was successfully field-tested at a depth of 10,900 m in the Mariana Trench, underscoring the potential of increased deep-ocean exploration through the development of additional soft robots that can function in conditions of extreme pressure (259).

#### High-performance slime: the Atlantic hagfish

The Atlantic hagfish (*Myxine glutinosa*) is an ancient species that is recognizable in fossils from 300 million years ago, and which is unique in the animal kingdom as having a skull but no spinal column (260). The hagfish’s defensive slime—which it produces in vast quantities within fractions of a second and is characterized by thousands of silklike protein threads (260)—has attracted curiosity for hundreds of years and transcended the scientific community to enter popular culture (261–263). A search of the World Intellectual Property Organization (WIPO) Patentscope database (264) in July 2022 found 1,170 patents that reference the hagfish, with applications including use of slime threads for high-performance fibers, safety helmets, bulletproof vests, and even antishark sprays (261, 262, 265, 266).

### Deep-sea sponges

#### Skeletons of the deep: deep-sea sponges

Deep-sea sponges have attracted intense interest, as they are known to form siliceous skeletons characterized by high levels of porosity that can act as natural tissue scaffolds (267–270). A challenge of treating bone defects in humans is the need for bone substitution materials, with the traditional sources being the patient themself (autogenous grafts), other individuals of the same species (allograft materials), or nonhuman species (xenografts) (271). The former is frequently not available, and the latter two carry risk of disease transmission and host rejection, posing a major biomedical challenge (271). Siliceous scaffolds provide an attractive source of either xenografts or biomimetic constructs inspired by their structure (268). A recent study found diversity in porosity and pore size among representative deep-sea sponge species (*Geodia barretti, Geodia atlantica, Stelletta normani, Phakellia ventilabrum*, and *Axinella infundibuliformis*), suggesting varying inspirations for tissue engineering applications (268).

High-definition flow simulations of the deep-sea sponge *Euplectella aspergillum* have shed light on the internal architecture of sponge skeletons and their capacity to reduce hydrodynamic stress (272), while other simulations of its grid-like structure and bracings resulted in biomimetic lattice geometries with implications for the design and resilience of modern infrastructure, and minimizing the amount of material needed for construction (215, 273, 274). One of the most iconic such examples is “The Gherkin” in London’s financial district, which due to its design is claimed to function on half the energy of a similarly sized office building (215). Similar structural analysis of the giant anchor spicules of *Monorhaphis chuni* have found optimized designs highly resistant to fractures and cracking, while simultaneously possessing optical properties enabling the transmissibility of visible light with potential fiber-optic applications (275, 276). The porous skeletons of deep-sea sponges have also inspired biomimetic design, with synthetic porous carbon fibers found to achieve high levels of absorption of oils and organic solvents, suggesting future use in bioremediation (277). Likewise the stiffness and toughness of sponge skeletons has inspired the development of novel multilayered pipewalls (278). Patent filings associated with deep-sea sponges include the production of a novel antifouling compound from *Geodia barretti* (Fig. 3D) (279), and a composite material based on the silicated collagen matrix of the glass rope sponge, *Hyalonema sieboldi* (280).

### Other deep-sea fauna

#### Moving rapidly between depths: the Ram’s horn squid

While squids do not have internal buoyancy mechanisms comparable to the swim bladders of fish, an unusual variation is found in the Ram’s horn squid (*Spirula spirula*), a deep-sea cephalopod mollusk that practices diel vertical migration, generally remaining at depths of 600 to 700 m during the day to avoid predation, and rising at night to depths of 100 to 300 m to feed (51, 281). The “Ram’s horn” of *S. spirula* comes from its capacity to manufacture a hollow gas-filled shell that remains almost entirely within its body, and which grows and adds chambers in an expanding spiral form throughout its life (Fig. 3E) (51). While fish swim bladders can equilibrate somewhat to differing depths and pressures (143, 282), this process is slow. The much more rapid diel vertical migration of *S. spirula* is mediated by its rigid shell (although the shells implode at depths of 1,500 m, marking a clear depth limit for the species) (51, 281). Anderson and co-authors note that the biomimetic design of the *Deepsea Challenger* manned submersible, which descended to the ocean’s deepest point (10,911 m) in 2012, drew on both the shape and vertical orientation of *S. spirula*, as well as its shell, which it mimicked with a low-density foam of hollow microballoons (15, 51, 283).

#### The finest protection: the Scaly-foot snail

The scaly-foot snail (*Chrysomallon squamiferum*) is a gastropod mollusk species that was discovered in 1999 and has since been found on three hydrothermal vent systems at depths of over 2,400 m (284). *C. squamiferum* is heavily armored, enabling it to survive the attacks of crabs that have been known to squeeze *C. squamiferum* in their claws for days (Fig. 3F) (285, 286). Similar to other mollusks, *C. squamiferum* has a multilayered shell, but its iron-infused outer layer is unique (derived from the mineral-rich vent fluids) and followed by a thick organic layer and a third stiff mineralized layer, yielding remarkable protection that has attracted the attention of the US Department of Defense, which has funded research to explore its biomimetic potential for armor development (285, 286).

#### Nontoxic antifouling: Streptomyces albidoflavus strains

The bacterium *S. albidoflavus* is ubiquitous, with strains isolated from sources as diverse as a golf course in Korea (287), a mangrove leaf in China (288), soil in Poland (289), and sediments collected at a depth of 5,100 m in the western Pacific Ocean (290). Noting the many novel compounds that have been identified in marine *Streptomyces*, the latter strain (*S. albidoflavus sp*. UST040711-291) was cultured, and a set of five structurally similar compounds were isolated and studied (290, 291). Testing of the *S. albidoflavus* compounds identified functional properties, associated with the 2-furanone ring, that delivered powerful antifouling outcomes, leading the research group to file a corresponding patent on associated nontoxic antifouling derivatives (290, 292).

#### Revitalizing potentials: cold-water corals

The cold-water coral *Lophelia pertusa* can be found at depths of up to 3,000 m and is extremely slow growing, with radiocarbon dating suggesting it can live for up to 1,000 y, and form reefs that have been dated at over 40,000 y old (293). It is highly vulnerable to deep-sea fishing gears (294) as well as oil exploration and extraction practices (295). Such environmental concerns have led to experimentation with *L. pertusa* to understand the impacts of sedimentation and discard of drill cuttings (296), as well as development of a biomimetic sensor that could monitor the deep-water sea-floor for impacts from drilling activities, using a system of cameras interspersed with nubbins and polyps of *L. pertusa* (297). Likewise, while deep-sea sponge skeletons have drawn the most active interest in the development of biomimetic tissue scaffolds for biomedical applications, the structure of deep-sea bamboo corals (*Isididae*) has noted potential as a bone implant or substrate for bone revitalization (86, 270, 298).

## A “forgotten” ocean economy sector: current challenges and future opportunities

As illustrated in this Review, the field of marine biomimetics is characterized by diversity: in the diversity of innovations it has generated, in the diversity of species that have provided inspiration (from megafauna to microorganisms) and in the diversity of ocean environments that sustain these species from the coastal zone to the deep sea. Furthermore, the economic value of innovations inspired by marine life is substantial (Box1). Other key components of biomimetics—imagination, imitation, and inspiration—are universal elements of the human experience, suggesting the potential for marine biomimetics as a productive activity across geographies.

Box 1. Examples of revenues and costs associated with key areas of marine biomimetics.

For comparison, estimated annual revenues of key ocean economy sectors include the offshore wind industry (USD 37 billion), cruise tourism (USD 47 billion), container shipping (USD 156 billion), seafood (276 billion), offshore oil and gas (USD 830 billion) (37)

- *Rust*. According to estimates, in Sweden alone, the annual costs associated with corrosion total approximately SEK 90 billion (USD 9.5 billion) (299). The development of biomimetic adhesive coatings, for instance with ceria nanoparticles inspired by mussel byssus threads, aim to cut corrosion rates (see the “Adhesion” section) (106).
- *Biofouling*. The global market for marine coatings is predicted to top USD 15 billion by 2024, and the economic losses associated with marine biofouling currently cost marine industries over USD 150 billion annually (108, 110, 111) (see the “Antifouling” section).
- *Tissue scaffolds*. In the United States alone, over 49 tissue-engineering companies have been established (including 21 companies in the commercial phase of development and generating sales of an estimated USD 9 billion annually in 2017 (300) (see the “Other application” and “Deep-sea sponges” sections).
- *Robotics*. The global underwater robotics market size is expected to reach USD 6.74 billion by 2025 (301), benefiting heavily from biomimetic design focused on the morphology and physiology of marine species (see the “Armor,” “Buoyancy,” “Movement,” “Deep-sea fishes,” and “Other deep-sea fauna” sections).
- *Pharmaceuticals*. Revenues from five “marine drugs” (FDA-approved pharmaceuticals with compounds derived, synthesized or inspired by naturally occurring marine natural products—Adcetris, Halaven, Lovaza, Prialt, and Yondelis) totaled over USD 12.1 billion from 2011 to 2020 (see the “Other applications” section and Supplementary Material).

Where diversity is less evident, however, is in the beneficiaries of marine biomimetics. The majority of innovations identified in this Review are associated with industries disproportionately headquartered in the world’s most highly industrialized countries: shipping, robotics, biomedical/biotechnology, wind energy, etc (33, 36). Similarly, the capacity to cover costs of developing innovations and to subsequently access relevant industry counterparts may be a particular challenge to ensuring these activities are inclusive and the benefits are equitably shared [e.g. the timeline for bringing a new pharmaceutical to market can stretch across decades with costs of up to USD 1 billion (26, 230)]. Although capacity building is a recognized priority of the UN Decade of Ocean Science for Sustainable Development 2021–2030 including for deep-sea research (302, 303), investments in marine science as well as contributions to the peer-reviewed literature remain highly skewed towards the world’s most highly industrialized countries (304).

Marine biomimetics has also been largely neglected in blue economy strategies and framings (33, 34, 49, 45–48, 50), and we suggest this may be related to the complexity and diversity of marine biomimetic applications as well as challenges in credibly valuing its economic benefits. While the economic value of innovations inspired by marine life can be estimated in some cases (Box1), relying solely on monetary valuations would be a missed opportunity for articulating the unique position marine biomimetics could play in the blue economy, and for understanding the full value of nature (40, 305). Marine biomimetics does not depend on continuous marine resource use or extraction, and often results in innovations that reduce pollution, energy loss, or emissions, giving it a vanishingly light footprint alongside the stomping footprints of conventional ocean industries like cruise tourism or offshore oil and gas extraction. This will position marine biomimetics as a useful illustration of a blue economy sector if it can ensure that it not only drives sustainability, but also equity and inclusivity, while also spurring greater support for funding basic research on the ocean and the life it contains.

Marine biomimetics is particularly reliant on access to well-functioning marine ecosystems and the capacity to study these. The degradation of marine ecosystems has been widely documented and reported (46, 306). Iconic marine ecosystems like tropical reefs, for instance, face an existential threat from climate change and increasingly frequent marine heatwaves (307), which are also a risk to deep-sea ecosystems (308). Similarly, some of the most remote and least-studied deep-sea environments on Earth have an uncertain future as proponents of mining the international seabed push towards commercial operations, as hydrocarbon extraction moves into deeper and riskier waters, and with continuing use of destructive fishing gears that persist even in purportedly protected areas (36, 42, 309, 310).

Can articulating the role and potential of marine biomimetics in the ocean economy prompt greater awareness of the benefits derived from the ocean? Can marine biomimetics be a sustainable ocean sector at the very heart of the blue economy, sparking inclusive and equitable collaborations across geographies, cultures, and disciplines? Can marine biomimetics become a clear illustration of the importance of nonmonetary valuation, spurring the protection of marine ecosystems as permanent repositories of inspiration? Can it inspire care, and ultimately a sense of stewardship? In this manuscript, we have illustrated the diverse scope and scale of marine biomimetics, and propose that working to address the challenges and questions in this section represents a rich research agenda and action space for scientists and policymakers alike.

## Notes

We define the ocean economy to be the sum of economic activities related to the ocean and its coasts. Some consider the blue economy to be largely synonymous with the ocean economy^49^, while others consider it to implicitly encompass additional dimensions such as sustainability^311^ and stewardship^312^. In this paper, we use this term to describe an aspirational vision of an ocean economy that is equitable, sustainable and inclusive, in line with articulations introduced around the 2012 UN Convention on Sustainable Development (Rio+20) emphasizing more equitable sharing of benefits^313–315^ and the Sustainable Blue Economy Finance Principles, which define a sustainable blue economy as one that “provides social and economic benefits for current and future generations; restores, protects and maintains diverse, productive and resilient ecosystems; and is based on clean technologies, renewable energy and circular material flows”^316^.

## Supplementary Material

pgac196_Supplemental_File
