# Supplementary material for: A forgotten element of the blue economy: marine biomimetics and inspiration from the deep sea
Source: PNAS Nexus. 2022 Sep 17;1(4):pgac196. doi: 10.1093/pnasnexus/pgac196 (PMC9802412; doi:10.1093/pnasnexus/pgac196)
Supplement: pgac196_Supplemental_File [file pgac196_supplemental_file.docx]

**SUPPLEMENTARY MATERIAL:**

**Sales information for marine drugs Adcetris ^TM^, Halaven ^TM^, Lovaza ^TM^, Prialt ^TM^, and Yondelis ^TM^**

(Accessed April 2022)

*Note: These five marine drugs are just a sampling of the full suite of those in clinical use for which sales figures were readily available (see also Papon et al. 2022 and Haque et al. 2022 for comprehensive reviews), and Papon et al. 2022 for market growth estimates for marine drugs.*

=================================

**Adcetris ^TM^**

**Reference**: <https://www.adcetris.com>

**Annual reports**: <https://www.seagen.com>

**Sales / licensing stats**:

2020 = USD 658.577 million / USD 126.756 million

2019 = USD 627.733 million / USD 138.491 million

2018 = USD 476.903 million / USD 83.440 million

2017 = USD 307.562 million / USD 66.056 million

2016 = USD 265.766 million / USD 67.455 million

2015 = USD 226.052 million / USD 40.980 million

2014 = USD 178.198 million / USD 40.004 million

2013 = USD 144.665 million / USD 17.818 million

2012 = USD 138.200 million / USD 5.065 million

2011 = USD 43.241 million

**Total sales 2011 – 2020:** USD 3,653.162 million

=================================

**Halaven ^TM^**

**Reference**: <http://www.halaven.com/metastatic-breast-cancer>

**Annual reports**: <https://www.eisai.com/index.html>

**Sales and licensing stats**:

2020 = JPY 37.6 billion

2019 = JPY 40.213 billion

2018 = JPY 41.289 billion

2017 = JPY 39.980 billion

2016 = JPY 37.328 billion

2015 = JPY 40.168 billion

2014 = JPY 35.314 billion

2013 = JPY 28.947 billion

2012 = JPY 22.611 billion

2011 = JPY 31.957 billion

**Total sales 2011 – 2020:** JPY 387.266 billion *(USD 3,126.024 million)*

=================================

**Lovaza ^TM^**

**Reference**: <https://lovaza.com>

**Annual reports**: <https://www.gsk.com/en-gb/home/>

**Sales and licensing stats**:

2020 N/A

2019 N/A

2018 N/A

2017 N/A

2016 = GBP 43 million

2015 = GBP 93 million

2014 = GBP 240 million / 41 million

2013 = GBP 584 million / 123 million

2012 = GBP 607 million / 445 million

2011 = GBP 569 million / 536 million

**Total sales 2011 – 2020:** GBP 3,281 million *(USD 4,290.753 million)*

=================================

**Prialt ^TM^**

**Reference:** <https://www.prialt.com>

**Annual reports:** <https://www.jazzpharma.com>

**Sales and licensing stats:**

2020 – N/A

2019 – N/A (TerSera Therapeutics acquired Prialt in 2018 for USD 80 million, but no annual report found)

2018 – USD 20.839 million

2017 – USD 27.361 million

2016 – USD 29.12 million

2015 – USD 26.44 million

2014 – USD 26.421 million

2013 – USD 27.103 million

2012 – USD 26.36 million

2011 – N/A

**Total sales 2011 – 2020:** USD 183.644 million

=================================

**Yondelis ^TM^**

**Reference**: <https://www.yondelis.com>

**Annual reports**: <https://pharmamar.com/en/>

**Sales and licensing stats**

2020 = EUR 73.3 million

2019 = EUR 71.880 million

2018 = EUR 73.835 million

2017 = EUR 84.6 million

2016 = EUR 88.2 million

2015 = EUR 88.4 million

2014 = EUR 69.637 million

2013 = EUR 74.350 million

2012 = EUR 66.080 million

2011 = EUR 74.112 million

**Total sales 2011 – 2020:** EUR 764.4 million *(USD 834.032 million)*

=================================

Total sales and licensing revenues for Adcetris, Halaven, Lovaza, Prialt, Yondelis

**Adcetris** USD 3,652.962 million

**Halaven** USD 3,126.024 million

**Lovaza** USD 4,290.753 million

**Prialt** USD 183.644 million

**Yondelis** USD 834.032 million

**TOTAL** USD 12.1 billion (2011-2020)
